# Global patterns and cause composition of adolescent acute respiratory failure–related mortality, 1990–2023

**DOI:** 10.5588/pha.26.0006

**Published:** 2026-05-18

**Authors:** Y.J. Han

**Affiliations:** Division of Pediatric Critical Care Medicine, Department of Pediatrics, Yonsei University College of Medicine, Seoul, South Korea.

**Keywords:** respiratory disease, global burden of disease, COVID-19, critical care, preventable death, population-level analysis

## Abstract

**BACKGROUND:**

Acute respiratory failure (ARF) is a life-threatening clinical syndrome that often reflects limitations in timely access to emergency and critical respiratory care. Among adolescents, severe respiratory illness remains an important but poorly characterised contributor to preventable mortality.

**OBJECTIVE:**

To describe long-term global trends and cause composition of adolescent ARF-related mortality, and assess changes associated with the coronavirus disease 2019 (COVID-19) pandemic. This was a descriptive analysis using a syndrome-based proxy derived from Global Burden of Disease 2023 cause-of-death estimates from 1990 to 2023.

**RESULTS:**

Between 1990 and 2019, global adolescent ARF proxy deaths declined modestly, followed by a sharp increase during the COVID-19 pandemic, peaking in 2021. Before 2020, lower respiratory infections accounted for over 85% of ARF proxy mortality, with a stable cause composition. During 2020–2021, COVID-19 emerged as a major contributor, accounting for nearly half of ARF proxy deaths at its peak. By 2023, total ARF proxy mortality and cause structure returned toward pre-pandemic patterns.

**CONCLUSION:**

Adolescent ARF-related mortality has declined modestly over three decades and shows a stable underlying structure re-emerging after the COVID-19 shock. These findings highlight persistent health-system vulnerabilities in delivering timely acute respiratory care for adolescents and support the need to strengthen emergency and critical care services alongside prevention.

Acute respiratory failure (ARF) is a common and life-threatening clinical syndrome in critical care, often requiring advanced respiratory support and timely access to effective emergency and critical care services. Among adolescents, severe respiratory failure contributes substantially to preventable mortality, reflecting not only biological susceptibility but also the capacity of health systems to deliver timely acute care. Despite its clinical importance, ARF is not represented as a discrete cause of death in population-level mortality frameworks, including the Global Burden of Disease (GBD) study, limiting direct assessment of its global mortality burden.^1,2^ At the population level, examination of ARF-related mortality therefore requires an indirect, syndrome-based perspective. The GBD study provides comprehensive and comparable estimates of mortality across countries and over time for a wide range of underlying causes of death,^1–5^ and has been instrumental in characterising global trends in major respiratory conditions such as lower respiratory infections and coronavirus disease 2019 (COVID-19).^1,2,6^ However, because GBD analyses are structured around etiologic categories rather than clinical syndromes, outcomes that converge on a shared final common pathway – such as severe respiratory failure – may be fragmented across causes and obscured when examined in isolation.^1,3,5^ Syndrome-based reconstruction offers a pragmatic approach to addressing this limitation by enabling examination of severe clinical outcomes that are not encoded as distinct causes of death. Similar strategies have been applied within the GBD framework to study other syndromic conditions, including sepsis, that likewise lack discrete cause-of-death classification.^7,8^ In this context, acute respiratory infections that commonly culminate in fatal respiratory deterioration provide a feasible basis for examining population-level patterns of ARF-related mortality.^1,2,9^

Adolescents represent a distinct and often under-recognised population within global health, occupying a transitional life stage characterised by evolving biological, behavioural, and health-system vulnerabilities. Yet, in many global burden analyses, adolescents are frequently aggregated with younger children or adults, constraining age-specific interpretation of severe respiratory outcomes and limiting insight into adolescent-specific risks.^1,2,10,11^ From a public health perspective, understanding the structure of ARF-related mortality among adolescents offers insight into persistent vulnerabilities of health systems to severe respiratory illness beyond pathogen-specific trends.

Framing ARF as a population-level indicator of acute-care–sensitive mortality may therefore provide a pragmatic lens for identifying gaps in timely emergency and critical care delivery during adolescence.

## METHODS

We conducted a descriptive analysis using a syndrome-based proxy approach to examine adolescent ARF-related mortality. Cause-of-death data were obtained from the GBD 2023 study, which provides internally consistent estimates of cause-specific mortality by age, sex, location, and year using standardised analytical methods.^1,2,12^ Data were accessed via the GBD 2023 Results Tool and covered the period from 1990 to 2023.

### Study population

The study population comprised adolescents aged 10–19 years of both sexes at the global level. Analyses were restricted to global aggregates, as the objective was to describe overall population-level patterns of ARF-related mortality rather than demographic or regional gradients.

### Operational definition of ARF proxy

Because ARF is not represented as a discrete cause of death within the GBD framework, ARF-related mortality was operationalised using a syndrome-based proxy. The proxy comprised a priori selected GBD cause-of-death categories most likely to result in life-threatening respiratory failure: lower respiratory infections, upper respiratory infections, pertussis, and COVID-19.^1,2,9,13^ Upper respiratory infections were included to capture severe upper airway conditions, such as croup or epiglottitis, that may precipitate ARF; mild or self-limited upper respiratory infections were not the focus of this proxy. The ARF proxy was intentionally restricted to acute infectious respiratory causes, reflecting the study’s focus on mortality arising from severe infections requiring timely diagnosis, referral, and access to critical respiratory care. Non-infectious causes of respiratory compromise, including drowning, trauma, poisoning, congenital anomalies, and chronic respiratory diseases, were excluded from the primary proxy definition but examined separately in sensitivity analyses.

### Outcome measures

The primary outcome was the annual number of deaths attributable to the infection-related ARF proxy. For each year, total ARF proxy deaths were calculated as the sum of cause-specific deaths across the four included categories. The secondary outcome was the cause composition of ARF proxy mortality, expressed as the proportion of total proxy deaths attributable to each cause within a given year. Cause composition in 2023 was further examined descriptively to assess concentration of causes in the most recent period.

### Statistical analysis

Analyses were descriptive. Annual trends in total ARF proxy mortality were summarised graphically. Temporal changes in cause composition were visualised using stacked bar plots to illustrate proportional contributions over time. Cause concentration in 2023 was assessed by ranking causes according to their proportional contribution to total ARF proxy deaths. No formal hypothesis testing or regression modelling was performed. Data processing and aggregation were conducted using standard spreadsheet software, and figures were generated using GraphPad Prism (GraphPad Software, San Diego, CA, USA). Although uncertainty intervals are available in GBD outputs, the present analysis focused on point estimates to describe overall temporal patterns and cause composition; uncertainty intervals were not displayed in the figures, as the objective was descriptive rather than inferential.

### Ethical statement

This study was based on secondary analysis of publicly available, de-identified data from the Global Burden of Disease Study. Ethical approval and informed consent were therefore not required.

## RESULTS

**FIGURE 1.**
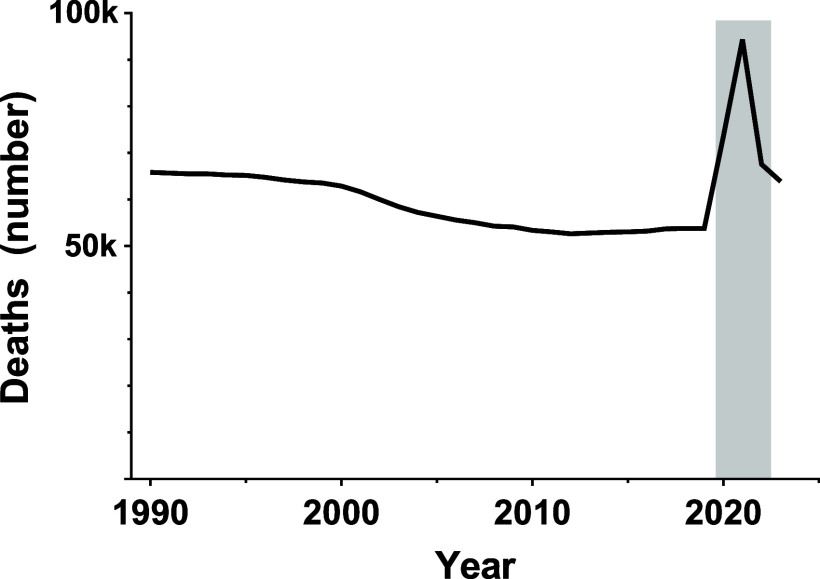
Global trends in adolescent ARF proxy mortality, 1990–2023. Annual deaths among adolescents aged 10–19 years were estimated using Global Burden of Disease 2023 cause-of-death data. ARF was operationalised as an infection-related, syndrome-based proxy derived from selected GBD underlying causes of death, comprising lower respiratory infections, upper respiratory infections, pertussis, and COVID-19, with total ARF proxy deaths calculated as the sum of cause-specific deaths. The shaded area indicates the COVID-19 pandemic period (2020–2022). ARF = acute respiratory failure; COVID-19 = coronavirus disease 2019.

Using this infection-related, syndrome-based construct, total ARF proxy deaths declined gradually at the global level between 1990 and 2019, decreasing from approximately 65,900 deaths in 1990 to 53,800 deaths in 2019 (Figure 1). This long-term trajectory was interrupted during the COVID-19 pandemic. In 2020, ARF proxy deaths increased substantially, followed by a pronounced peak in 2021, when total deaths reached approximately 94,400, the highest level observed during the study period. Subsequently, ARF proxy mortality declined sharply in 2022 and 2023, returning toward pre-pandemic levels.

### Cause composition of ARF proxy mortality over time

**FIGURE 2.**
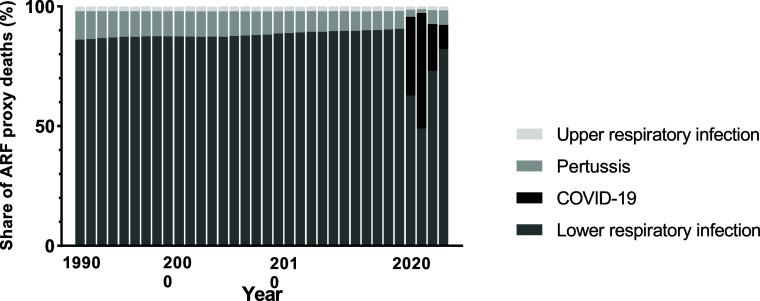
Temporal changes in the cause composition of adolescent ARF proxy mortality, 1990–2023. Stacked bars show the proportion of total ARF proxy deaths attributable to lower respiratory infections, COVID-19, pertussis, and upper respiratory infections for each year. ARF was operationalised as an infection-related, syndrome-based proxy derived from selected Global Burden of Disease underlying causes of death. ARF = acute respiratory failure; COVID-19 = coronavirus disease 2019.

Throughout the pre-pandemic period (1990–2019), the cause composition of ARF proxy mortality among adolescents remained highly stable (Figure 2; Supplementary Data Table S1). Lower respiratory infections accounted for the majority of ARF proxy deaths, representing more than 85% of total proxy mortality in most years, while pertussis and upper respiratory infections contributed smaller but persistent proportions. COVID-19 was absent from the cause structure prior to 2020. During the pandemic period (2020–2021), the cause composition changed substantially. In 2020, COVID-19 emerged as a major contributor, accounting for approximately one third of ARF proxy deaths. This contribution increased further in 2021, when COVID-19 accounted for nearly half of total ARF proxy mortality, while remaining slightly lower than the contribution of lower respiratory infections. By 2022 and 2023, the relative contribution of COVID-19 declined markedly.

### Cause concentration in the most recent year

**FIGURE 3.**
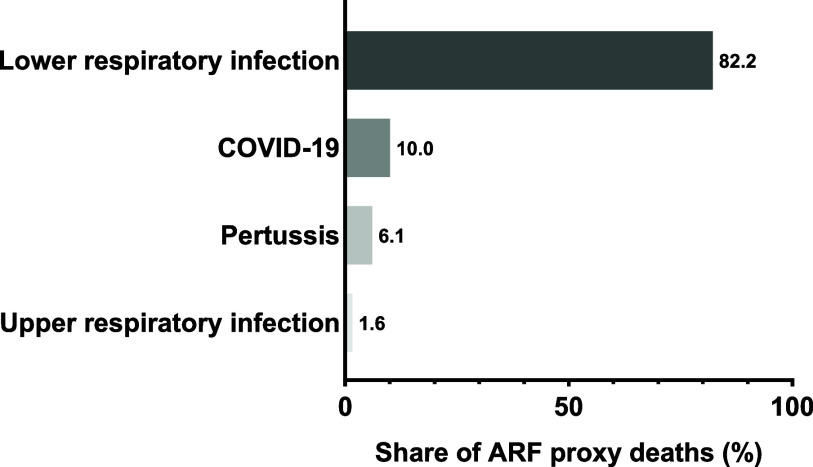
Cause composition of adolescent ARF proxy mortality in 2023. Proportional contributions of infection-related causes to ARF proxy mortality among adolescents aged 10–19 years in 2023. The ARF proxy included lower respiratory infections, COVID-19, pertussis, and upper respiratory infections, representing infection-related causes of death within the Global Burden of Disease framework. Bars indicate the percentage of total ARF proxy deaths attributable to each cause. ARF = acute respiratory failure; COVID-19 = coronavirus disease 2019.

In 2023, ARF proxy mortality among adolescents was concentrated in a small number of causes (Figure 3; Table). Lower respiratory infections accounted for over 80% of total ARF proxy deaths (82.2%), followed by COVID-19 (10.0%), pertussis (6.1%), and upper respiratory infections (1.6%). Together, the two leading causes (lower respiratory infections and COVID-19) accounted for more than 90% of ARF proxy mortality.

**TABLE.** Cause-specific deaths and composition of adolescent acute respiratory failure (ARF) proxy mortality in 1990–2023.

Cause	1990 deaths, n	% of total ARF proxy deaths	2023 deaths, n	% of total ARF proxy deaths
Lower respiratory infections	56,710	86.1	52,507	82.2
COVID-19	0	0.0	6,417	10.0
Pertussis	7,860	11.9	3,892	6.1
Upper respiratory infections	1,309	2.0	1,050	1.6
Total ARF proxy	65,879	100.0	63,866	100.0

ARF was operationalised as a syndrome-based proxy comprising lower respiratory infections, upper respiratory infections, pertussis, and COVID-19. Percentages indicate the proportion of total ARF proxy deaths attributable to each cause in the specified year. COVID-19 was absent prior to 2020 and is shown as 0 for 1990 for completeness. Deaths were rounded from Global Burden of Disease estimates; percentages may not sum to exactly 100% due to rounding.

COVID-19 = coronavirus disease 2019.

### Sensitivity analysis including selected non-infectious respiratory causes

As a sensitivity analysis, pulmonary aspiration and foreign body in the airway were added to the infection-related ARF proxy to assess the robustness of the proxy definition (Supplementary Data Table S2). Across most years, inclusion of these non-infectious respiratory causes increased total ARF proxy deaths by approximately 7%–9% relative to the infection-related proxy alone. During the COVID-19 peak in 2020–2021, the relative contribution of pulmonary aspiration and foreign body deaths was lower, reflecting the marked increase in COVID-19–related ARF proxy mortality.

## DISCUSSION

Using GBD 2023 cause-of-death estimates, adolescent mortality attributable to an infection-related ARF proxy derived from underlying GBD causes of death was examined from 1990 to 2023.^1^ Three main findings emerged. First, despite substantial global progress in infectious disease prevention and critical care, mortality declined only modestly from 1990 to 2019.^1,2^ Second, the COVID-19 pandemic produced a marked but time-limited increase in deaths in 2020–2021, altering short-term cause composition without displacing lower respiratory infections as the leading contributor.^1,2^ Third, by 2023, the overall cause structure had largely returned to its pre-pandemic configuration, suggesting the persistence of an underlying pattern of severe respiratory mortality in adolescence rather than clear evidence of a sustained structural shift.^1,2^

The modest long-term reduction over more than three decades contrasts with substantial changes observed in global mortality patterns during the same period.^1,2,10,11,14^ Acute respiratory infections continue to account for a large share of global respiratory deaths in GBD analyses.^1,2,9,13^ These findings are consistent with the possibility that further reductions during adolescence may be influenced not only by pathogen-specific advances but also by timely access to effective acute and critical care.^1,2,14^ The COVID-19 pandemic represented an unprecedented disruption to global respiratory mortality.^1,2,10,11^ COVID-19 rapidly emerged as a major contributor, driving the sharp increase observed in 2020–2021. Rather than replacing existing causes, these deaths were added to a pre-existing structure.^1,2^ By 2023, although COVID-19 remained present, the overall composition resembled the pre-pandemic pattern, suggesting re-alignment following resolution of the acute pandemic phase.^1,2,6^

The ARF proxy represents a grouping of underlying causes of death that converge on a shared final pathway of severe respiratory deterioration. Proportional comparisons across individual categories should therefore be interpreted cautiously. GBD cause-of-death categories differ in scope and granularity, and COVID-19 reflects a distinct classification introduced during the study period.^1,2,9,13^ Accordingly, the primary interpretive value of this analysis lies in examining stability and disruption of the overall proxy structure over time, rather than in direct comparisons between individual causes. Although external clinical validation was beyond the scope of this population-level analysis, the use of standardised GBD estimates and consistency across sensitivity analyses support the robustness of the observed mortality patterns.

Sensitivity analyses incorporating selected acute non-infectious respiratory causes, including pulmonary aspiration and foreign body in the airway, resulted in a consistent 7%–9% increase in total ARF proxy mortality across most years. Inclusion of these causes did not materially alter long-term trends or the overall structural patterns observed. During the COVID-19 peak, their relative contribution declined, reflecting a denominator effect associated with the surge in COVID-19–related mortality.^1,2^ These findings indicate that the principal conclusions are not driven by exclusion of acute non-infectious respiratory causes.^1,2^

By reconstructing ARF-related mortality using a syndrome-based proxy, this study illustrates how population-level analyses can capture severe respiratory outcomes not directly observable within cause-specific mortality frameworks.^1,7,8^ The persistence of ARF proxy mortality among adolescents may reflect limitations of pathogen-specific progress in settings where timely access to acute respiratory and critical care remains uneven. Strengthening early recognition, referral pathways, and surge capacity may therefore represent important components of broader efforts to reduce avoidable respiratory deaths in this age group.

This study has several limitations. First, ARF proxy mortality was inferred from cause-of-death classifications rather than direct clinical diagnoses, and misclassification is possible. Second, while GBD provides uncertainty intervals for cause-specific mortality estimates, these were not incorporated into graphical displays, as the objective was to describe broad structural patterns rather than to make comparative statistical inferences. Third, the proxy was intentionally restricted to infection-related causes and does not capture the full spectrum of non-infectious ARF aetiologies. Fourth, analyses were limited to global aggregates and did not examine regional or demographic gradients. Finally, estimates for the most recent years may be influenced by reporting delays and residual effects of the COVID-19 pandemic. These limitations are inherent to global burden analyses and do not detract from the utility of the findings for describing population-level patterns.

## CONCLUSION

Using a syndrome-based proxy derived from GBD cause-of-death data, this study demonstrates that adolescent ARF-related mortality has declined only modestly over the past three decades and exhibits a stable underlying structure that re-aligned toward its pre-pandemic pattern following the COVID-19 shock. These findings underscore the continued global burden of life-threatening respiratory failure among adolescents and support the need for population-level strategies that strengthen timely access to acute and critical respiratory care alongside disease-specific interventions.

## Supplementary Material

## References

[1] GBD 2023 Causes of Death Collaborators. Global burden of 292 causes of death in 204 countries and territories and 660 subnational locations, 1990–2023: a systematic analysis for the Global Burden of Disease Study 2023. Lancet. 2025;406(10513):1811-1872. 41092928 10.1016/S0140-6736(25)01917-8 PMC12535838

[2] GBD 2021 Causes of Death Collaborators. Global burden of 288 causes of death and life expectancy decomposition in 204 countries and territories and 811 subnational locations, 1990–2021: a systematic analysis for the Global Burden of Disease Study 2021. Lancet. 2024;403(10440):2100-2132. 38582094 10.1016/S0140-6736(24)00367-2 PMC11126520

[3] Murray CJL, Lopez AD. Measuring the global burden of disease. N Engl J Med. 2013;369(5):448-457. 23902484 10.1056/NEJMra1201534

[4] Murray CJ, Lopez AD. Global mortality, disability, and the contribution of risk factors: Global Burden of Disease Study. Lancet. 1997;349(9063):1436-1442. 9164317 10.1016/S0140-6736(96)07495-8

[5] Murray CJL. The Global Burden of Disease Study at 30 years. Nat Med. 2022;28(10):2019-2026. 36216939 10.1038/s41591-022-01990-1

[6] Chow EJ, Uyeki TM, Chu HY. The effects of the COVID-19 pandemic on community respiratory virus activity. Nat Rev Microbiol. 2023;21(3):195-210. 36253478 10.1038/s41579-022-00807-9 PMC9574826

[7] GBD 2021 Global Sepsis Collaborators. Global, regional, and national sepsis incidence and mortality, 1990-2021: a systematic analysis. Lancet Glob Health. 2025;13(12):e2013-e2026. 41135560 10.1016/S2214-109X(25)00356-0

[8] Rudd KE, Global, regional, and national sepsis incidence and mortality, 1990-2017: analysis for the Global Burden of Disease Study. Lancet. 2020;395(10219):200-211. 31954465 10.1016/S0140-6736(19)32989-7 PMC6970225

[9] GBD 2021 Lower Respiratory Infections and Antimicrobial Resistance Collaborators. Global, regional, and national incidence and mortality burden of non-COVID-19 lower respiratory infections and aetiologies, 1990-2021: a systematic analysis from the Global Burden of Disease Study 2021. Lancet Infect Dis. 2024;24(9):974-1002. 38636536 10.1016/S1473-3099(24)00176-2 PMC11339187

[10] GBD 2021 Demographics Collaborators. Global age-sex-specific mortality, life expectancy, and population estimates in 204 countries and territories and 811 subnational locations, 1950-2021, and the impact of the COVID-19 pandemic: a comprehensive demographic analysis for the Global Burden of Disease Study 2021. Lancet. 2024;403(10440):1989-2056. 38484753 10.1016/S0140-6736(24)00476-8 PMC11126395

[11] GBD 2023 Demographics Collaborators. Global age-sex-specific all-cause mortality and life expectancy estimates for 204 countries and territories and 660 subnational locations, 1950-2023: a demographic analysis for the Global Burden of Disease Study 2023. Lancet. 2025;406(10513):1731-1810. 41092927 10.1016/S0140-6736(25)01330-3 PMC12535839

[12] Murray CJL, GBD 2010: design, definitions, and metrics. Lancet. 2012;380(9859):2063-2066. 23245602 10.1016/S0140-6736(12)61899-6

[13] GBD 2016 Lower Respiratory Infections Collaborators. Estimates of the global, regional, and national morbidity, mortality, and aetiologies of lower respiratory infections in 195 countries, 1990-2016: a systematic analysis for the Global Burden of Disease Study 2016. Lancet Infect Dis. 2018;18(11):1191-1210. 30243584 10.1016/S1473-3099(18)30310-4 PMC6202443

[14] World Health Organization. World health statistics 2025: monitoring health for the SDGs, sustainable development goals. Geneva: WHO, 2025.

