# Supplementary material for: Global patterns and cause composition of adolescent acute respiratory failure–related mortality, 1990–2023
Source: Public Health Action. 2026 May 18;16(2):81–5. doi: 10.5588/pha.26.0006 (PMC13182887; doi:10.5588/pha.26.0006)
Supplement: Supplementary file 1 [file pha26-0006_supplementarydata1.pdf]

Supplementary Materials

These supplementary tables provide detailed annual cause-specific estimates underlying the analyses of adolescent acute respiratory failure (ARF) proxy mortality presented in the main manuscript. Supplementary Table S1 reports yearly deaths and cause composition for the infection-related ARF proxy from 1990 to 2023. Supplementary Table S2 presents sensitivity analyses incorporating selected non-infectious respiratory causes to assess the robustness of the proxy definition. All estimates were derived from Global Burden of Disease (GBD) 2023 cause-of-death data for adolescents aged 10–19 years.

Supplementary Table S1. Cause-specific deaths and composition of adolescent acute respiratory failure (ARF) proxy mortality, 1990–2023

Year	Lower respiratory infections		COVID-19		Pertussis		Upper respiratory infections		Total ARF proxy deaths,
	n	%	n	%	n	%	n	%	n
1990	56711	86.1	0	0.0	7860	11.9	1308	2.0	65879
1991	56782	86.4	0	0.0	7653	11.6	1295	2.0	65729
1992	56905	86.8	0	0.0	7353	11.2	1283	2.0	65541
1993	57074	87.1	0	0.0	7188	11.0	1278	1.9	65539
1994	56993	87.3	0	0.0	7037	10.8	1270	1.9	65300
1995	56897	87.3	0	0.0	7046	10.8	1263	1.9	65206
1996	56643	87.5	0	0.0	6871	10.6	1257	1.9	64771
1997	56191	87.5	0	0.0	6765	10.5	1256	2.0	64212
1998	55865	87.5	0	0.0	6725	10.5	1262	2.0	63852
1999	55580	87.4	0	0.0	6730	10.6	1266	2.0	63575
2000	54967	87.4	0	0.0	6659	10.6	1260	2.0	62886
2001	53838	87.3	0	0.0	6615	10.7	1247	2.0	61699
2002	52493	87.4	0	0.0	6333	10.5	1228	2.0	60054
2003	51085	87.3	0	0.0	6223	10.6	1202	2.1	58509
2004	50135	87.6	0	0.0	5919	10.3	1181	2.1	57236
2005	49529	87.8	0	0.0	5736	10.2	1161	2.1	56427
2006	48939	88.0	0	0.0	5531	9.9	1142	2.1	55613
2007	48509	88.2	0	0.0	5392	9.8	1117	2.0	55019
2008	48180	88.7	0	0.0	5025	9.3	1093	2.0	54298
2009	48132	88.9	0	0.0	4955	9.1	1074	2.0	54160
2010	47626	89.1	0	0.0	4751	8.9	1057	2.0	53434
2011	47382	89.3	0	0.0	4592	8.7	1060	2.0	53034
2012	47090	89.4	0	0.0	4540	8.6	1062	2.0	52692
2013	47362	89.7	0	0.0	4408	8.3	1031	2.0	52801
2014	47582	89.8	0	0.0	4405	8.3	1028	1.9	53015
2015	47610	89.8	0	0.0	4344	8.2	1091	2.1	53046
2016	47963	90.0	0	0.0	4232	7.9	1077	2.0	53272
2017	48429	90.1	0	0.0	4244	7.9	1060	2.0	53733
2018	48634	90.4	0	0.0	4131	7.7	1047	1.9	53813
2019	48736	90.6	0	0.0	4025	7.5	1016	1.9	53778
2020	46216	62.8	24194	32.9	2155	2.9	1036	1.4	73602
2021	46245	49.0	45445	48.2	1631	1.7	1030	1.1	94351
2022	49433	73.2	13146	19.5	3963	5.9	1028	1.5	67569
2023	52507	82.2	6418	10.0	3892	6.1	1050	1.6	63866

Acute respiratory failure (ARF) was operationalized as a syndrome-based proxy comprising lower respiratory infections, upper respiratory infections, pertussis, and coronavirus disease 2019 (COVID-19). Percentages indicate the proportion of total ARF proxy deaths in each year. Estimates were derived from Global Burden of Disease 2023 cause-of-death data for adolescents aged 10–19 years.

ARF = acute respiratory failure; COVID-19 = coronavirus disease 2019.

Supplementary Table S2. Sensitivity analysis including selected non-infectious respiratory causes of adolescent acute respiratory failure (ARF) proxy mortality

Year	Infection-related ARF proxy deaths, n	Pulmonary aspiration / foreign body in airway deaths, n	Expanded ARF proxy deaths, n	Additional deaths as % of infection-related proxy
1990	65879	4888	70767	7.4
1991	65729	4910	70639	7.5
1992	65541	4956	70497	7.6
1993	65539	5014	70554	7.7
1994	65300	5044	70344	7.7
1995	65206	5055	70261	7.8
1996	64771	5064	69836	7.8
1997	64212	5019	69231	7.8
1998	63852	5037	68889	7.9
1999	63575	5102	68677	8.0
2000	62886	5049	67935	8.0
2001	61699	5006	66706	8.1
2002	60054	4954	65008	8.2
2003	58509	4880	63389	8.3
2004	57236	4820	62056	8.4
2005	56427	4756	61183	8.4
2006	55613	4695	60307	8.4
2007	55019	4645	59664	8.4
2008	54298	4568	58866	8.4
2009	54160	4482	58642	8.3
2010	53434	4460	57894	8.3
2011	53034	4438	57472	8.4
2012	52692	4463	57155	8.5
2013	52801	4499	57300	8.5
2014	53015	4515	57530	8.5
2015	53046	4540	57586	8.6
2016	53272	4616	57888	8.7
2017	53733	4668	58400	8.7
2018	53813	4693	58506	8.7
2019	53778	4740	58518	8.8
2020	73602	4704	78306	6.4
2021	94351	4846	99197	5.1
2022	67569	5086	72654	7.5
2023	63866	5311	69177	8.3

Infection-related ARF proxy mortality was defined using deaths attributable to acute respiratory infections within the Global Burden of Disease (GBD) cause-of-death framework. As a sensitivity analysis, deaths due to pulmonary aspiration and foreign body in the airway were additionally included to assess the robustness of the proxy definition. Expanded ARF proxy deaths represent the sum of infection-related ARF proxy deaths and pulmonary aspiration/foreign body deaths. "Additional deaths as % of infection-related proxy" was calculated as deaths from pulmonary aspiration or foreign body in the airway divided by infection-related ARF proxy deaths for each year.

ARF = acute respiratory failure.
